# Genome Sequence of a *Helicobacter pylori* Strain Isolated from a Mexican Patient with Intestinal Gastric Cancer

**DOI:** 10.1128/genomeA.01214-13

**Published:** 2014-01-23

**Authors:** Alfonso Méndez-Tenorio, Violeta Larios-Serrato, Gabriela Edith Olguín-Ruiz, Carlos Javier Sánchez-Vallejo, Roberto Carlos Torres-López, Francisco Avilés-Jiménez, Margarita Camorlinga-Ponce, Javier Torres

**Affiliations:** a Escuela Nacional de Ciencias Biológicas of Instituto Politécnico Nacional, Distrito Federal (DF), Mexico; b Unidad de Investigación en Enfermedades Infecciosas, Hospital de Pediatría, Centro Médico Nacional Siglo-XXI, Instituto Mexicano del Seguro Social (IMSS), DF, Mexico

## Abstract

*Helicobacter pylori* strains are the major risk factor for gastric cancer. Strains vary in their content of disease-associated genes, so genome-wide analysis of cancer-isolated strains will help elucidate their pathogenesis and genetic diversity. We present the draft genome sequence of *H. pylori* isolated from a Mexican patient with intestinal gastric cancer.

## GENOME ANNOUNCEMENT

*Helicobacter pylori*, which is a class I carcinogen (1) and the first bacterial infection associated with human cancer, is responsible for almost 80% of all distal gastric cancer (GC). This disease is the second leading cause of death from all cancers worldwide and its incidence is estimated to be increasing because of aging of populations in middle- and low-income regions (2). However, <3% of all *H. pylori*-infected individuals will ever develop GC, because strains vary in their content of disease-associated genes. Genome diversity applies to disease organism as well as human populations since *H. pylori* has coevolved with human races for millennia (3, 4). The study of whole-genome sequences from strains isolated from GC patients is necessary to better understand the pathogenesis of the disease and to identify virulence genes useful in the screening of patients with higher risk.

We report the draft sequence of the genome of an *H. pylori* strain (CG-IMSS-2012) isolated from a 57-year-old Mexican man diagnosed with gastric cancer of the intestinal type. This genome sequence will add to the very scarce genome sequences reported from clinical isolates and will provide valuable information for a better understanding of GC, particularly in Latin America, a region with the highest GC mortality rates worldwide. The *H. pylori* CG-IMSS-2012 strain was sequenced using the 454 FLX platform (Roche, Germany), generating a library containing 309,265 single reads with an average length of 209 bp and a 35-fold average coverage. The draft genome sequence was generated by the combination of a *de novo* assembly using the Newbler gsAssembler (release 1.1.03.24) and reference assembly using the AMOS assembler (version 3.1.0). This strategy provided 47 large contigs (45 of them longer than 200 bp). This draft genome sequence has a size of about 1,599,050 bp and a G+C content of 39.0%. As a reference, we employed the genome sequence of *H. pylori* strain ELS37 (NC_017063). The contigs were ordered using this sequence because previous analysis showed a high degree of similarity between these two strains. The contigs were then remapped with AMOS assembler (5). Contig annotation was performed with the NCBI Prokaryotic Genome Automatic Annotation Pipeline (PGAAP) version 2.0 (6). The genome revealed 91.5% of coding regions and 1,613 genes, including 39 RNA genes. The average length for protein-coding genes was found to be 929 bp. Major virulence markers were annotated, such as *cagA*, *vacA*, and several genes belonging to the *cag* pathogenicity island. With PCR, we confirmed the presence of genes *cagA* (including typing of the repeating EPIYA motifs), *vacA* (typing of s and m regions), *babA*, and *oipA*. Genome and gene comparison analysis (7) by MLST with other fully and partially sequenced strains demonstrates that this genome is phylogenetically closer to the *H. pylori* ELS37 and to other Latin America strains.

### Nucleotide sequence accession numbers.

The 45 annotated contig sequences of the *H. pylori* CG-IMSS-2012 genome were deposited in the WGS database of the NCBI and they are available through the accession numbers AWUL00000001 through AWUL00000045.
